# Antimicrobial Peptides from Rat-Tailed Maggots of the Drone Fly *Eristalis tenax* Show Potent Activity against Multidrug-Resistant Gram-Negative Bacteria

**DOI:** 10.3390/microorganisms8050626

**Published:** 2020-04-25

**Authors:** Rolf Hirsch, Jochen Wiesner, Armin Bauer, Alexander Marker, Heiko Vogel, Peter Eugen Hammann, Andreas Vilcinskas

**Affiliations:** 1 Fraunhofer Institute for Molecular Biology and Applied Ecology, Department of Bioresources, Ohlebergsweg 12, 35392 Giessen, Germany; rolf.hirsch@evotec.com (R.H.); jochen.wiesner@biochemie.med.uni-giessen.de (J.W.); 2 Evotec International GmbH, Marie-Curie-Str. 7, 37079 Göttingen, Germany; peter.hammann@evotec.com; 3 Sanofi-Aventis Deutschland GmbH, Industriepark Höchst, 65926 Frankfurt, Germany; Armin.Bauer@sanofi.com (A.B.); alexander.marker@sanofi.com (A.M.); 4 Max-Planck Institute for Chemical Ecology, Department of Entomology, Hans-Knoell-Strasse 8, 07745 Jena, Germany; hvogel@ice.mpg.de; 5 Institute for Insect Biotechnology, Justus Liebig University of Giessen, Heinrich-Buff-Ring 26-32, 35390 Giessen, Germany

**Keywords:** antimicrobial peptides, Gram-negative bacteria, antibiotic, innate immunity, transcriptomics, *Eristalis tenax*

## Abstract

The spread of multidrug-resistant Gram-negative bacteria is an increasing threat to human health, because novel compound classes for the development of antibiotics have not been discovered for decades. Antimicrobial peptides (AMPs) may provide a much-needed breakthrough because these immunity-related defense molecules protect many eukaryotes against Gram-negative pathogens. Recent concepts in evolutionary immunology predict the presence of potent AMPs in insects that have adapted to survive in habitats with extreme microbial contamination. For example, the saprophagous and coprophagous maggots of the drone fly *Eristalis tenax* (Diptera) can flourish in polluted aquatic habitats, such as sewage tanks and farmyard liquid manure storage pits. We used next-generation sequencing to screen the *E. tenax* immunity-related transcriptome for AMPs that are synthesized in response to the injection of bacterial lipopolysaccharide. We identified 22 AMPs and selected nine for larger-scale synthesis to test their activity against a broad spectrum of pathogens, including multidrug-resistant Gram-negative bacteria. Two cecropin-like peptides (EtCec1-a and EtCec2-a) and a diptericin-like peptide (EtDip) displayed strong activity against the pathogens, even under simulated physiological conditions, and also achieved a good therapeutic window. Therefore, these AMPs could be used as leads for the development of novel antibiotics.

## 1. Introduction

The increasing prevalence of multidrug-resistant pathogens combined with the declining approval rate for new antibiotics has created an urgent need for novel compounds to fuel the drug development pipeline. As an alternative to the screening of chemical libraries, antimicrobial peptides (AMPs) have emerged as a promising source of new drugs, because these effector molecules of the innate immune system represent an evolutionarily-conserved pan-eukaryotic defense system against bacterial pathogens [1]. AMPs impose weaker selection pressure than conventional antibiotics and could, therefore, achieve long-lasting effectiveness [2]. Insects produce a remarkably diverse repertoire of AMPs that can be screened for their activity against human pathogens [3].

The selection of certain insect species for the knowledge-based discovery of potent AMPs was inspired by the hypothesis that the most promising species are those that have adapted to survive in habitats with extreme microbial contamination [4]. For example, the burying beetle *Nicrophorus vespilloides* feeds and reproduces on carcasses and, thus, produces a larger number of AMPs than most beetles [5,6]. Similarly, the rat-tailed maggots of the drone fly *Eristalis tenax* have been introduced as a model of ecological immunology, because they have adapted to survive in aquatic habitats with extreme microbial loads, such as sewage tanks and manure pits. An initial suppression subtractive hybridization screen identified 19 putative inducible AMPs in this species [7]. We sought to expand the number of candidate AMPs by using next-generation sequencing to analyze the immunity-related transcriptome of rat-tailed maggots in a systematic manner. The comparison of untreated maggots and those that were injected with bacterial lipopolysaccharide (LPS) to elicit a strong immune response revealed 22 transcripts encoding putative AMPs. For further analysis, we selected three cecropin-like peptides of the sarcotoxin subclass (EtCec1, EtCec2, and EtCec3), which feature a conserved C-terminal glycine residue that is thought to undergo post-translational amidation [8,9]. We synthesized both the non-amidated and amidated versions of each AMP (Table 1). In addition, we selected a diptericin-type AMP (EtDip) and two defensin-like AMPs (EtDef1 and EtDef4). These nine *E. tenax* AMPs were tested against an extended panel of Gram-negative clinical isolates in order to determine their toxicity, therapeutic potential, mode of action, and potential to confer selective pressure for resistance.

## 2. Materials and Methods 

### 2.1. RNA-Seq and de novo Transcriptome Assembly

Last-instar *E. tenax* larvae were injected with microbial LPS, as previously described [7]. Untreated control larvae were maintained under the same conditions. After 8 h, whole larvae were flash frozen and then pulverized in liquid nitrogen. RNA was extracted from each specimen using the Direct-Zol RNA MiniPrep kit with a DNase step (Zymo Research, Irvine, CA, USA). The quantity of extracted RNA was determined using a NanoDrop ND-1000 spectrophotometer (Thermo Fisher Scientific, Waltham, MA, USA) and RNA integrity was confirmed while using an RNA Nanochip on a 2100 Bioanalyzer (Agilent Technologies, Santa Clara, CA, USA). Poly(A) mRNA enrichment, TrueSeq RNA library generation, and sequencing on an Illumina HiSeq 2500 instrument was carried out at the Max Planck Genome Centre, yielding ~30 million paired-end (2 × 100 bp) reads for each sample. The sequence reads were clipped for remaining adapters, quality trimmed, and combined for *de novo* assembly using CLC Genomics Workbench v9.1 (Qiagen, Venlo Netherlands). The transcriptome was annotated using BLAST, Gene Ontology, and InterProScan in the Blast2GO software suite as previously described [5]. Protein and signal peptide prediction was followed by the identification of conserved and hypothetical AMPs using our standard pipeline [11]). All of the putative AMPs were screened using the CAMPR3 (Collection of Antimicrobial Peptides) AMP-prediction tool (http://www.camp3.bicnirrh.res.in/predict/; [12]).

### 2.2. Synthetic Peptides

Table 1 lists he amino acid sequences of the peptides used in this study. The peptides were prepared by solid-phase synthesis (GenScript, Piscataway, NJ, USA) on a polymeric carrier resin and they were analyzed by reversed-phase chromatography while using an Alltech Alltima C18 4.6 × 250 mm column (Thermo Fisher Scientific) with an ascending acetonitrile gradient in water containing 0.05–0.065% trifluoroacetic acid. The peptides were detected and quantified by UV absorption at 220 nm and by electrospray ionization mass spectrometry (ESI-MS). The purity of each peptide was at least 90%.

### 2.3. Strains and Culture Conditions

The strains that were used in this study (Table 2 and Table S1) were cultivated at 37 °C in cation-adjusted Mueller–Hinton broth (CAMB) in Erlenmeyer flasks, or on the same medium with agar in Petri dishes. Exceptionally, *Mycobacterium smegmatis* was cultivated at 37 °C in brain heart infusion (BHI) medium that was supplemented with 1% Tween-80 in Erlenmeyer flasks, or on the same medium lacking detergent but with agar in Petri dishes. Strains listed with Robert Koch Institute (RKI) strain numbers are clinical isolates from the RKI strain collection originating from hospitalized patients in Germany. The remaining strains originated from the Sanofi strain collection and they were originally purchased from the American Type Culture Collection or the German Collection of Microorganisms and Cell Cultures. Meropenem/colistin-resistant strains were maintained, as described above, with the appropriate antibiotic added at levels below the minimal inhibitory concentration (MIC).

### 2.4. Inhibition of Microbial Growth

The MIC values were determined in 384-well microtiter plates with an assay volume of 20 µL. Immediately before each assay, the cultures of test organisms (bacteria grown for 18 h, *Candida albicans* and *M. smegmatis* grown for 48 h) were diluted with the appropriate medium to a density of 1 × 10^6^ cells/mL (*C. albicans*), 1 × 10^5^ cells/mL (*M. smegmatis*) or 5 × 10^5^ cells per mL (other species). The peptides were dissolved in sterile water at a concentration of 10.24 mg/mL and a 1:2 dilution series was prepared in a 384-well microtiter plate to achieve the concentration range 10,240–0.3125 µg/mL in a volume of 50 µL. We transferred 2-µL aliquots from this dilution series to new 384-well microtiter plates while using a CyBio-Well 384-channel automated pipettor (Analytic Jena, Jena, Germany) and added 18 µL of the diluted suspension of each test species using a Viafill reagent dispenser (Integra), thus reducing the final peptide concentration 10-fold to the range 1024–0.03125 µg/mL. The cells were incubated at 37 °C and 85% relative humidity, shaking at 180 rpm for 48 h (*M. smegmatis*), 24 h (*C. albicans*) or 18 h (other species). The growth of *M. smegmatis* was measured using the BacTiter-Glo luminescence test system (Promega, Madison, WI, USA). The growth of the other species was determined by visual inspection of the microtiter plate and turbidimetry based on the optical density at 600 nm (OD_600_). The MIC was defined as the peptide concentration at which no bacterial growth was observed. We supplemented the test medium with either 150 mM NaCl or 1.25 mM CaCl_2_ to represent monovalent and divalent cations to simulate physiological conditions. Bacteria for synergy testing were diluted to 5 × 10^5^ cells/mL in CAMB containing sub-MIC concentration of the antibiotics and/or peptides to investigate potential interactions with the clinical antibiotics meropenem, gentamicin, tobramycin, tetracycline, tigecycline, rifampicin, and colistin.

### 2.5. Checkerboard Assay

The interactions between pairs of compounds were analyzed using checkerboard assays in a 384-well microtiter plate with an assay volume of 20 µL. Immediately before the test runs, the bacterial cultures were diluted in CAMB to a density of 5 × 10^5^ cells/mL. The two compounds for combinatorial testing were arrayed as 10-fold concentrated serial dilutions with respect to the final concentration, vertically for one compound and horizontally for the other, in the same 384-well microplate. Subsequently, we transferred 2-µL aliquots to new 384-well microtiter plates, and growth inhibition in each well was determined, as described for the MIC values above. Fractional inhibitory concentration (FIC) and FIC_index_ values for each combination were calculated using the following formulae, where a 

FIC_index_ ≤0.5 indicates synergy and a FIC_index_ >4 indicates antagonism.

FIC for compound A = MIC of compound A in combination/MIC of compound A

FIC for compound B = MIC of compound B in combination/MIC of compound B

FIC_index_ = FIC A + FIC B

### 2.6. Serial-passage Mutagenesis

EtCec1-a was dissolved in sterile water to obtain a 10-fold concentrated stock solution (512 µg/mL), allowing for us to prepare a 1:2 dilution series in a 384-well microtiter plate to cover the concentration range 5210–0.16 µg/mL in a volume of 50 µL. We added gentamicin and colistin in the concentration range 320–0.01 µg/mL as controls. From this dilution series, 2-µL aliquots were transferred to new 384-well microtiter plates using the CyBio-Well 384-channel automated pipettor, and 18 µL of an *Escherichia coli* (ATCC 25922) suspension containing 5 × 10^5^ cells/mL was added to each well while using the Viafill reagent dispenser to achieve final peptide concentrations in the range 512–0.016 µg/mL. The plates were incubated at 37 °C and 85% relative humidity, shaking at 180 rpm for 24 h before measuring the MIC by visual inspection and turbidimetry (OD_600_), as above. The second-highest concentration of EtCec1-a allowing for bacterial growth was diluted 1:10,000 in fresh CAMB and added to a new assay plate containing a dilution series of EtCec1-a. This serial passaging was consecutively repeated for 30 days.

### 2.7. Hemolysis of Human Erythrocytes

Fresh citrated stabilized human whole blood was centrifuged at 500× *g* for 5 min. and the cell sediment was washed three times with phosphate buffered saline (PBS) before resuspending in PBS (50-fold the initial sample volume) to generate the human red blood cell (hRBC) suspension. The peptides were dissolved in sterile water at a concentration of 10.24 mg/mL and a dilution series was prepared in a 384-well microtiter plate, as above, to achieve the concentration range 10,240–0.3125 µg/mL in a volume of 50 µL. Triton X-100 (positive control) was diluted in sterile water to cover the concentration range 20–0.02%. We transferred 5-µL aliquots to new 384-well microtiter plates using the CyBio-Well 384-channel automated pipettor and added 45 µL of the hRBC suspension while using the Viafill reagent dispenser to achieve the final peptide concentration range 1024–0.031 µg/mL. After incubation at 37 °C. for 3 h, the plate was centrifuged and 20 μL of the supernatant from each well was transferred to a new microtiter plate. The quantity of released hemoglobin was determined by measuring the absorbance at 540 nm using a Lumistar plate reader (BMG Labtech, Ortenberg, Germany). The percent hemolysis was calculated by comparing to a control without peptide (no hemolysis) and a control with 2% Triton X-100 (100% hemolysis).

### 2.8. Cytotoxicity Assay Based on ATP Quantification

The cytotoxicity of the AMPs was determined in 96-well microtiter plates with an assay volume of 200 µL. The peptides were dissolved in PBS and a 1:2 dilution series in DMEM-F12 medium (supplemented with 1% nonessential amino acids, 1% sodium pyruvate, and 5% heat-inactivated fetal calf serum) was prepared in nine steps to produce test concentrations of 400, 200, 100, 25, 12.5, 6.25, 3.13, and 1.56 μM (1655–6.46 µg/mL for EtCec1-a, 1713–6.68 µg/mL for EtCec2-a, 1622–6.33 µg/mL for EtCec3-a, 2661–10.38 µg/mL for EtDip). Stocks of the mycoplasma-free HepG2 cell line HB-8065 (ATCC) stored in liquid nitrogen were seeded at a density of 20,000 cells/well into 96-well microplates in 100 μL DMEM-F12 supplemented as above. After incubation overnight at 37 ± 1 °C in a 5% CO_2_ atmosphere, 100 μL of the test solution was added per well in six replicates. Ketoconazole was used as a positive control and PBS as a negative control. The plates were incubated for 24 h, as above, and the cell viability was assessed using the luminescent CellTiter-Glo ATP monitoring kit (Promega) to determine the quantity of ATP. We determined half-maximal cytotoxic concentrations (IC_50_) and no observed effect concentrations (NOEC), the latter being defined as the highest peptide concentration that showed no cytotoxic effect (cell viability >80%) or peptide precipitation.

### 2.9. Cytotoxicity Assay Based on Neutral Red Uptake

HepG2 cells and peptides were prepared as described above, and cell viability was assessed based on the ability of lysosomes to store the dye neutral red (Sigma–Aldrich, ST. Louis, MO, USA). The cells were incubated for 3 h with a neutral red solution, washed twice, and lysed. After complete lysis, the quantity of neutral red was determined by measuring the absorption at 540 nm while using a Tecan Genios Pro plate reader.

### 2.10. Interaction with the Human ERG (ether-a-go-go related gene) Potassium Channel

The interaction of the peptides with the human ERG (hERG) potassium channel was analyzed using the automated patch-clamp method [13]. Chinese hamster ovary (CHO) hERG Duo cells constitutively expressing hERG (B’SYS) were diluted to 8 × 10^6^ cells/mL in Ex-Cell CHO medium (Sigma–Aldrich) containing 25 mM HEPES, 100 U/mL penicillin-streptomycin, and 0.004% soybean trypsin inhibitor. The peptides were diluted from a stock concentration of 10 mM in DMSO to final concentrations of 0.12, 0.37, 1.1, 3.3, 10, and 30 μM (0.5–124 µg/mL for -1a, 0.51–128 µg/mL for EtCec2-a, 0.49–122 µg/mL for EtCec3-a, 0.8–200 µg/mL for EtDip) in extracellular medium (150 mM NaCl, 4 mM KCl, 2 mM CaCl_2_, 1 mM MgCl_2_, 10 mM HEPES, 10 mM glucose) containing 0.06% Pluronic F-68 and 0.3% residual DMSO. The response of hERG to each peptide was assessed while using a QPatch HTX station and QPlates (Sophion/Biolin Scientific, Ballerup, Denmark) by recording the tail current following channel repolarization. Half-maximal inhibitory concentrations (IC_50_) were determined from a series of six concentrations applied to the cells in ascending order. Terfenadine citrate was used as a positive control and extracellular medium as a negative control.

### 2.11. Plasmastability

EtCec1-a, EtCec2-a, and EtDip were dissolved as 1 mM stock solutions in sterile deionized water and then incubated in 500 µL plasma from different species (human, mouse and rat) to a final concentration of 5 µM (20.7, 21.4, and 33.3 µg/mL for EtCec1-a, EtCec2-a, and EtDip, respectively) at 37 °C. At different time points (0, 1, 4, and 24 h), 100-µL samples were transferred to 500 µL ethanol containing 0.5% (*v/v*) NH_3_, and the plasma proteins were precipitated by centrifugation for 20 min. at 1735× *g*. EtCec1-a, EtCec2-a, and EtDip (10 µL injection volume) were analyzed in triplicate by LC-MS, and their stability was evaluated by comparing samples of with plasma incubated timepoints (t1, t4, t24) to untreated samples (t0). The peptides were separated on an AERIS Peptide 3.6-µm XB-C18 50 × 2.1 mm column (Phenomenex, Torrance, CA, USA) with an ascending acetonitrile gradient in water (that was supplemented with 0.1% formic acid) at a flow rate of 500 µL/min.

### 2.12. Metabolic Stability

The metabolic stability of the *E. tenax* peptides was assessed by determining the intrinsic clearance while using cryopreserved human hepatocytes in suspension culture in a volume of 60 µL with a seeding densitiy of 0.5 × 10^6^ hepatocytes/mL. The peptides were added at a concentration of 1 µM (4.14, 4.28, and 6.65 µg/mL for EtCec1-a, EtCec2-a, and EtDip, respectively). Scaled hepatic clearance for human was calculated based on a weight of 25.71 g liver/kg [14] and a hepatocellularity of 99 × 10^6^ cells/g liver [15].

## 3. Results

### 3.1. Transcriptome Assembly and AMP Identification

The transcriptome of *E. tenax* maggots was assembled *de novo* based on 61 million sequence reads with an average length of 97 bp after trimming, resulting in 44,882 contigs (minimum contig size = 200 bp) with an N50 contig size of 930 bp, a maximum contig length of 13,350 bp, and a GC content of 47.8%. We identified 22 conserved AMP genes, 16 of which were upregulated in LPS-challenged insects and six of which were downregulated. All 22 sequences returned BLAST hits matching other insect AMPs. We focused on a subset of five upregulated and one downregulated AMPs for functional characterization, representing three of the four larger AMP families in *E. tenax* (Figures S1 and S2). We selected the two most upregulated cecropin-like peptides EtCec1, EtCec2, and the downregulated EtCec3, the most upregulated diptericin-like peptide EtDip, and the two defensin-like peptides EtDef1 and EtDef4. The two defensins were selected based on their strong upregulation representing the highest (EtDef4) and the lowest (EtDef1) expressed AMP within the defensin family. We also prepared amidated versions of the cecropin-like peptides (EtCec1-a, EtCec2-a, and EtCec3-a) making nine test peptides in total (Table 1). Figure S2 depicts sequence similarities to representative peptide homologues for each of the three AMP families.

### 3.2. Antimicrobial Activity against Reference Strains

We tested EtCec1, EtCec2, EtCec3, their amidated counterparts, and EtDip against a panel of Gram-positive bacteria (*Staphylococcus aureus* ATCC 25923, *S. aureus* ATCC 33592, *Staphylococcus epidermidis* ATCC 35984, *Enterococcus faecium* DSM 17050 and *Listeria monocytogenes* DSM 20600), Gram-negative bacteria (*E. coli* D31, *E. coli* ATCC 25922, *Klebsiella pneumoniae* DSM 30104, *Acinetobacter baumannii* ATCC 19606, *Pseudomonas aeruginosa* ATCC 27853, and *Proteus mirabilis* DSM 4479), in addition to the acid-fast bacterium *M. smegmatis* ATCC 607 and the yeast *C. albicans* FH2173. All of the peptides lacked any substantial activity (MIC ≥1024 µg/mL) against Gram-positive bacteria, *M. smegmatis*, and *C. albicans*, except EtDip, which showed weak activity (MIC = 64 µg/mL) against *M. smegmatis*. There was no activity against *P. mirabilis* (MIC >1024 µg/mL), which shows intrinsic resistance to cationic peptides [16,17]. Only EtCec1-a showed activity against *P. aeruginosa* (MIC = 32 µg/mL), but this peptide also showed higher activity (MIC 4–8 µg/mL) against the strains of *E. coli*, *K. pneumoniae* and *A. baumannii* we tested. EtCec2-a showed moderate activity against the same strains (MIC = 8–32 µg/mL). For both of these peptides, the loss of the C-terminal amide increased the MIC two-fold. EtCec3 and EtDip showed no activity against the test strains (Table 2). The defensins EtDef1 and EtDef4 were tested against the same test strains, although *E. coli* D31 was replaced with *Micrococcus luteus* DSM 20030. EtDef4 showed moderate activity against *S. aureus*, *L. monocytogenes*, *M. smegmatis*, and *M. luteus* (MIC = 16–32 µg/mL), whereas EtDef1 was inactive against the test strains. Neither of the defensins showed significant activity against the Gram-negative bacteria, *S. epidermidis*, *E. faecium*, or against the yeast *C. albicans* (MIC >128 µg/mL) (Table 2).

### 3.3. Activity against an Extended Panel of Gram-Negative Clinical Isolates

In order to investigate the activity of EtCec1-a and EtCec2-a in more detail, we tested them against a panel of clinical isolates, including *E. coli* (26 strains), *Enterobacter cloacae* (23 strains), *Enterobacter aerogenes* (synonym *Klebsiella aerogenes*, one strain), *K. pneumoniae* (21 strains), *Klebsiella oxytoca* (two strains), *Salmonella enterica* (10 strains), *Citrobacter freundii* (one strain), *A. baumannii* (20 strains), *Acinetobacter pittii* (one strain), *P. aeruginosa* (two strains), *Stenotrophomonas maltophilia* (two strains), *Morganella morganii* (four strains), and *Seratia fonticola* (one strain). The isolates were selected based on their resistance phenotype, including resistance to colistin, and the presence of genes encoding different types of carbapenemases (Table S1). We determined the MIC of colistin and meropenem (a frequently-used carbapenem) in parallel with the AMP testing in order to confirm the resistance phenotype. EtCec1-a was active against the isolates of *E. coli*, *Enterobacter*, *Klebsiella*, *S. enterica*, *C. freundii*, and *Acinetobacter*, with MICs in the range 2–32 µg/mL (Table S2). In agreement with the preliminary results, only low activity (MIC = 128 µg/mL) was observed against the *P. aeruginosa* isolates. EtCec1-a showed little or no activity (MIC ≥ 64 µg/mL) against *S. maltophilia*, *M. morganii*, and *S. fonticola*, in accordance with previous findings that these species, like *P. mirabilis*, are resistant to cationic peptides [18]. The MIC of EtCec2-a for most of the isolates of *E. coli*, *Enterobacter*, *Klebsiella*, and *Acinetobacter* ranged from 4 to ≥128 µg/mL (Table S3). In contrast to EtCec1-a, most isolates of *S. enterica* and *C. freundii* were insensitive to EtCec2-a (MIC ≥128 µg/mL). No activity was observed against the isolates of *M. morganii* and *S. fonticola*, as expected and reported for EtCec1-a (MIC >128 µg/mL). The *P. aeruginosa* and *S. maltophilia* isolates were also insensitive to EtCec2-a.

We also calculated the MIC_50_ and MIC_90_ values for *E. coli*, *E. cloacae*, *K. pneumoniae*, *S. enterica,* and *A. baumannii*. MIC_50_ values confirmed that EtCec1 was 8–32 times more active than EtCec2 against *E. cloacae*, *K. pneumoniae*, and *S. enterica*, four times more active against *E. coli*, and twice as active against *A. baumannii*. Similar results were observed for the MIC_90_ values (Table 3).

**Figure 1:**
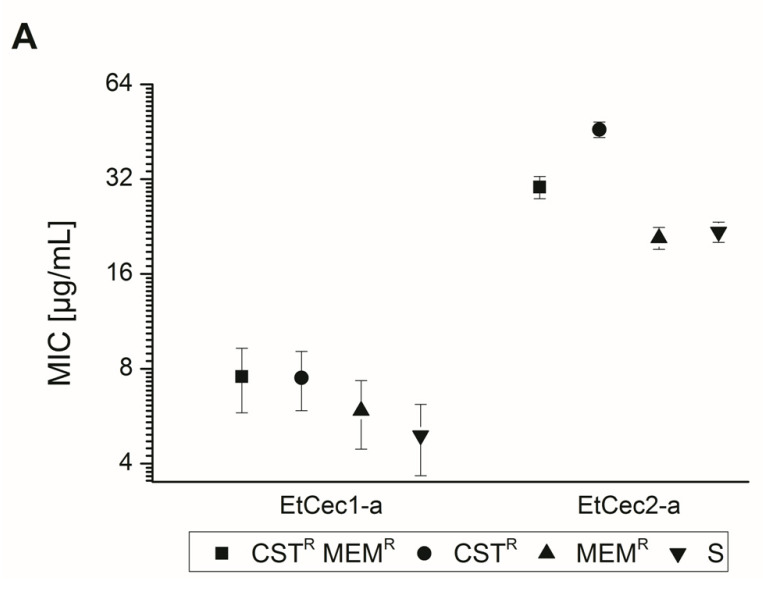
Activity of EtCec1-a and EtCec2-a against colistin/meropenem-resistant (CST^R^MST^R^; squares), colistin-resistant (CST^R^; circles), meropenem-resistant (MST^R^; triangles), and colistin/meropenem-sensitive (S, inverted triangles) isolates. Mean MIC values are shown for 12 colistin/meropenem-resistant, 21 colistin-resistant, 34 meropenem-resistant, and 27 colistin/meropenem-sensitive isolates with 99% confidence intervals.

We compared the signed rank median MIC values of EtCec1-a and EtCec2-a for 12 colistin/meropenem-resistant, 21 colistin-resistant, 34 meropenem-resistant, and 27 colistin/meropenem-sensitive isolates to test for a correlation between colistin and/or meropenem resistance and reduced sensitivity to AMPs. The intrinsically colistin-resistant *Stenotrophomonas*, *Morganella*, and *Serratia* strains and all *Salmonella* strains were excluded for this analysis. No major differences were observed for the activity of EtCec1-a against these isolates (Figure 1). EtCec2-a displayed lower activity against the colistin-resistant isolates, which reached significance within the 99% confidence intervals (Figure 1).

### 3.4. Impact of the C-terminal Amidation

We also tested the non-amidated peptides EtCec1 and EtCec2 against the full panel of clinical isolates in addition to the C-terminally amidated peptides, which most likely represent the mature AMPs produced by the *E. tenax* larvae (Table S1). The loss of C-terminal amidation was generally associated with lower antibacterial activity, although the severity of this effect differed among the isolates. The MIC_50_ and MIC_90_ values confirmed the higher activity of the peptides with C-terminal amides (Table 3).

### 3.5. Activity under Simulated Physiological Conditions

The antibacterial activity of some AMPs is compromised by high salt concentrations [19,20]. Therefore, we determined the MIC values of EtCec1 and EtCec2 against selected bacterial strains under standard conditions in CAMB and in parallel in the same medium that was supplemented with 150 mM NaCl or 1.25 mM CaCl_2_, approximately representing the salt concentration in human plasma. Although this did reduce the activity of both peptides, the effect was small and it was not observed in all of the test strains (Table 4). The salt-dependent increase in MIC typically did not exceed two-fold, with the exception of EtCec1 tested against one strain of *K. pneumoniae*, where the MIC was four times higher. 

### 3.6. Antibacterial Activity in Combination with Approved Antibiotics 

We carried out preliminary experiments to determine the activity of meropenem, gentamicin, tobramycin, tetracycline, tigecycline, rifampicin, and colistin against *E. coli* ATCC 25922 in the presence of sub-MIC concentrations of EtCec1-a, EtCec2-a, EtCec1-a, and EtDip in order to investigate whether the *E. tenax* AMPs can synergize with or potentiate the activity of established antibiotics. Only the activity of colistin appeared to be markedly improved by the peptides.

Next we determined the MICs of EtCec1, EtCec2, EtCec3, their amidated counterparts, and EtDip in the presence of sub-MIC concentrations of colistin (0.075 µg/mL) against *E. coli* ATCC25922, *E. coli* RKI 131/08, *K. pneumoniae* DSM 30104, *K. pneumoniae* RKI 93/10, *K. oxytoca* RKI 5207, *E. cloacae* RKI 146/09, *A. baumannii* ATCC 19606, and *A. baumannii* RKI 19/09. The MICs of all the peptides were strongly reduced in the presence of 0.075 µg/mL colistin, although the effects differed among the peptides and test strains. The activity of EtCec1 was potentiated 2–8-fold by colistin against *E. coli*, *E. cloacae,* and the *Klebsiella* and *A. baumannii* isolates (MIC = 2–8 µg/mL), whereas the activity of EtCec1-a was potentiated 2–4-fold (MIC = 2–4 µg/mL). The susceptibility of the test strains to EtCec2 was increased 2–16-fold (MIC = 2–16 µg/mL), although its activity against *E. cloacae* was unaffected. The MIC of EtCec2-a was reduced 8–16-fold for *E. coli* and the *Klebsiella* strains (MIC = 2–8 µg/mL), but there was no influence on its activity against *E. cloacae* and the *A. baumannii* strains. EtCec3, EtCec3-a and EtDip showed greatly reduced MICs of 4 µg/mL for *E. coli* and two *Klebsiella* strains, representing a minimum 256-fold increase in activity (Table 5). The activity of gentamicin, tetracycline, and meropenem was unaffected by the presence of sub-MIC concentrations of colistin. We also tested the interaction of colistin with EtDef1 and EtDef4, revealing that the MICs for each peptide were reduced 32-fold (MIC = 4 µg/mL). 

**Figure 2:**
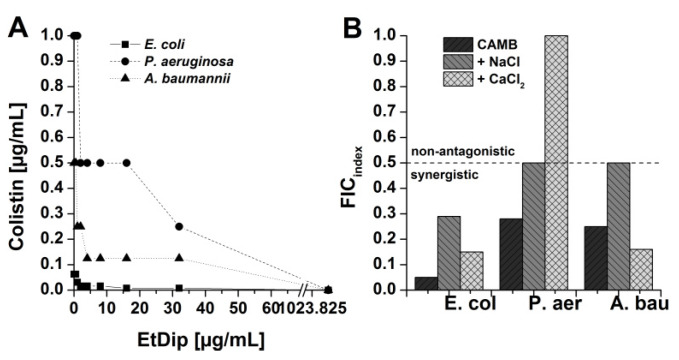
Interaction between EtDip and colistin. (**A**) Checkerboard assay against *E. coli* ATCC 25922, *P. aeruginosa* ATCC 27853 and *A. baumannii* ATCC 19606 in cation-adjusted Mueller-Hinton broth (*CAMB*) depicted as an isobologram. (**B**) Calculated FIC_index_ (fractional inhibitory concentration index) values against *E. coli*, *P. aeruginosa* (*P. aer.*), and *A. baumannii* (*A. bau.*) in CAMB, CAMB adjusted to 150 mM NaCl *(+NaCl*), and CAMB adjusted to 1.25 mM CaCl_2_ *(+CaCl_2_*). FIC_index_ values ≤0.5 indicate synergy.

We investigated the interaction between EtDip and colistin in more detail using a checkerboard assay against *E. coli* ATCC25922, *A. baumannii* ATCC19606, and *P. aeruginosa* ATCC 27853. Checkerboard analysis was carried out in CAMB and in parallel in CAMB adjusted to 150 mM NaCl or 1.25 mM CaCl_2_ in order to include the impact of physiological salt concentrations on the EtDip–colistin interaction. Synergistic interactions against *E. coli, A. baumannii,* and *P. aeruginosa* were observed under all conditions with the exception of *P. aeruginosa* in the presence of CaCl_2_ (Figure 2).

### 3.7. Preliminary Qualitative SAR Studies on Interaction of AMPs with Polymyxin Derivatives

We tested the effects of sub-MIC concentrations of eight different polymyxin B (PMB) derivatives on the activity of EtCec1-a, EtCec2-a, EtCec1-a, EtDip, EtDef1, and ETDef4 in order to investigate the interaction between the *E. tenax* AMPs and colistin (Table 6). Whereas different derivatives displayed various interaction patterns with the AMPs, only PMB displayed synergistic interactions with all of the AMPs (MIC = 2 µg/mL). Three PMB derivatives potentiated the activity of EtDef4: A000173039A was active against *E. coli* at 8 µg/mL, but reduced the MIC of EtDef4 from >256 to 64 µg/mL at a concentration of 0.25 µg/mL; A000173031A showed activity against *E. coli* at 64 µg/mL, but reduced the MIC of EtDef4 to 16 µg/mL at a concentration of 4 µg/mL; and, A000160918 was inactive against *E. coli* but reduced the MIC of EtDef4 to 32 µg/mL at a concentration of 4 µg/mL (it also increased the MIC of EtCec1-a from 16 to 128 µg/mL). Polymyxin B nonapeptide (PMBN) and two further derivatives also decreased the susceptibility of *E. coli* to EtCec1-a (MIC = 64–128 µg/mL). The derivative A000173033A did not affect the activity of the *E. tenax* peptides. We also tested seven different polymyxin E (colistin) derivatives (Table 6). Four derivatives had no impact on the MIC of the AMPs, but colistins E1 and E2 and derivative A000500146A were active against *E. coli* at a concentration of 1 µg/mL. Only colistin E2 demonstrated synergy with all of the *E. tenax* AMPs, reducing the MIC to 2–4 µg/mL. Colistin E1 only affected the activity of EtCec1-a, EtCec2-a and EtDef4 (reducing the MIC to 4–8 µg/mL). Colistin E1 did not interact with EtCec3-a, EtDip or EtDef1. Derivative A000500146A affected the activity of all peptides, except EtDef1. The activity of colistin, meropenem, gentamicin, and tetracycline was not affected by the polymyxin derivatives.

### 3.8. Hemolytic Activity 

**Figure 3:**
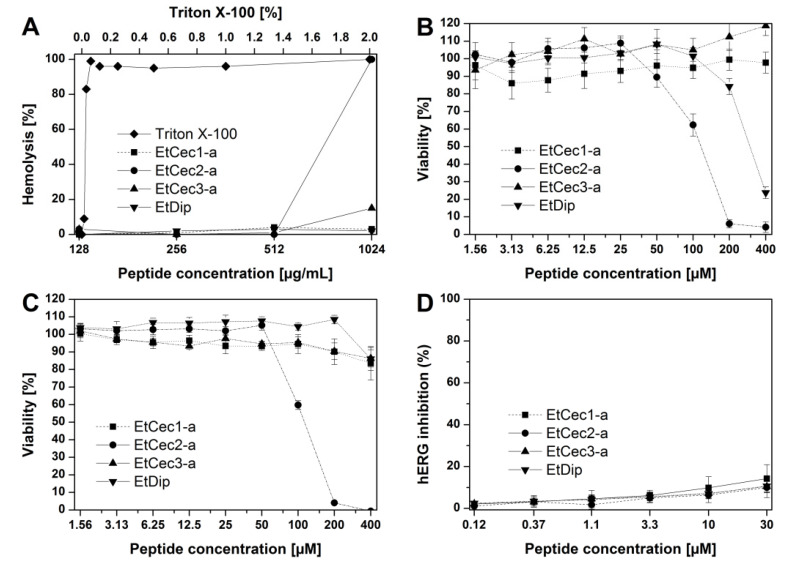
Toxicity of the *Eristalis tenax* AMPs EtCec1-a, EtCec2-a, EtCec3-a and EtDip. (**A**) Hemolytic activity in the presence of human erythrocytes. (**B**) Cytotoxic effects against HepG2 cells evaluated by neutral red uptake. (**C**) Cytotoxic effects against HepG2 cells evaluated by ATP concentration. (**D**) Cardiotoxic effects against the human ERG potassium channel.

We tested the hemolytic activity of EtCec1-a, EtCec2-a, EtCec3-a, and EtDip by investigating their ability to disrupt the membrane of human erythrocytes and, thus, release hemoglobin to evaluate the potential of the *E. tenax* AMPs for systemic applications (Figure 3). The minimal hemolytic concentration of EtCec2-a was 512 µg/mL, and the other peptides showed no hemolytic activity even at the highest tested concentration of 1024 µg/mL.

### 3.9. Toxicity Studies 

The therapeutic window (NOEC/MIC) of the AMPs was determined by testing the cytotoxicity of EtCec1-a, EtCec2-a, EtCec3-a, and EtDip against HepG2 human hepatocellular carcinoma cells, specifically by measuring the intracellular ATP concentration and the ability of the lysosomes to take up the dye neutral red. EtCec1-a, EtCec3-a, and EtDip were non-cytotoxic, with NOECs greater than 1655, 1622, and 1330 µg/mL, corresponding to 400, 400, and 200 µM, respectively (Figure 3). EtCec2-a was moderately toxic (NOEC = 535 µg/mL or 125 µM). We then investigated the cardiotoxicity of the peptides by measuring the effect on hERG. This voltage gated potassium channel is important for the repolarization of the cardiac action potential, and functional disruption can cause fatal ventricular tachyarrhythmia (torsade de pointes) [13,21]. No target-specific activity was observed. The IC_50_ values of EtCec1-a, EtCec2-a, EtCec3-a, and EtDip were greater than 124, 128, 121, and 200 µg/mL, respectively, corresponding to >30 µM (Figure 3).

### 3.10. In vitro Stability Studies 

**Figure 4:**
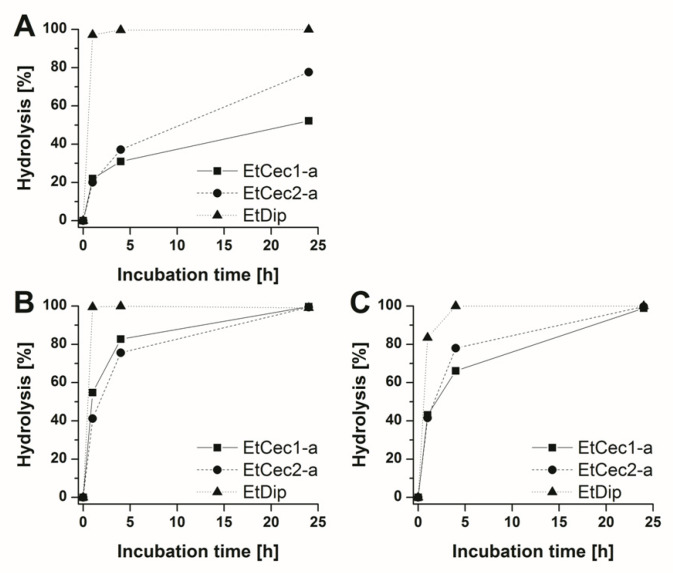
Percent hydrolysis of the *Eristalis tenax* AMPs EtCec1-a, EtCec2-a and EtDip after incubation in plasma from (**A**) human, (**B**) mouse, and (**C**) rat (*n* = 3).

The metabolic stability of the *E. tenax* peptides was determined by measuring the intrinsic clearance in human hepatocytes and calculating the scaled hepatic clearance as well as the half-life. EtCec1-a, EtCec2-a, and EtDip were found to be intrahepatic stable, with half-lives of 428, 206, and 345 min., respectively. We next measured the clearance of EtCec1-a, EtCec2-a, and EtDip from human, mouse, and rat plasma. All of the peptides were found to be unstable in plasma. EtCec1-a and EtCec2-a were completely hydrolyzed in mouse and rat plasma within 24 h, whereas 48% of EtCec1-a and 22% of EtCec2-a remained in human plasma after 24 h (Figure 4A,B). EtDip was rapidly hydrolyzed in all of the plasma samples, with ≥83% hydrolysis after 1 h (Figure 4C).

### 3.11. Development of Resistance 

**Figure 5:**
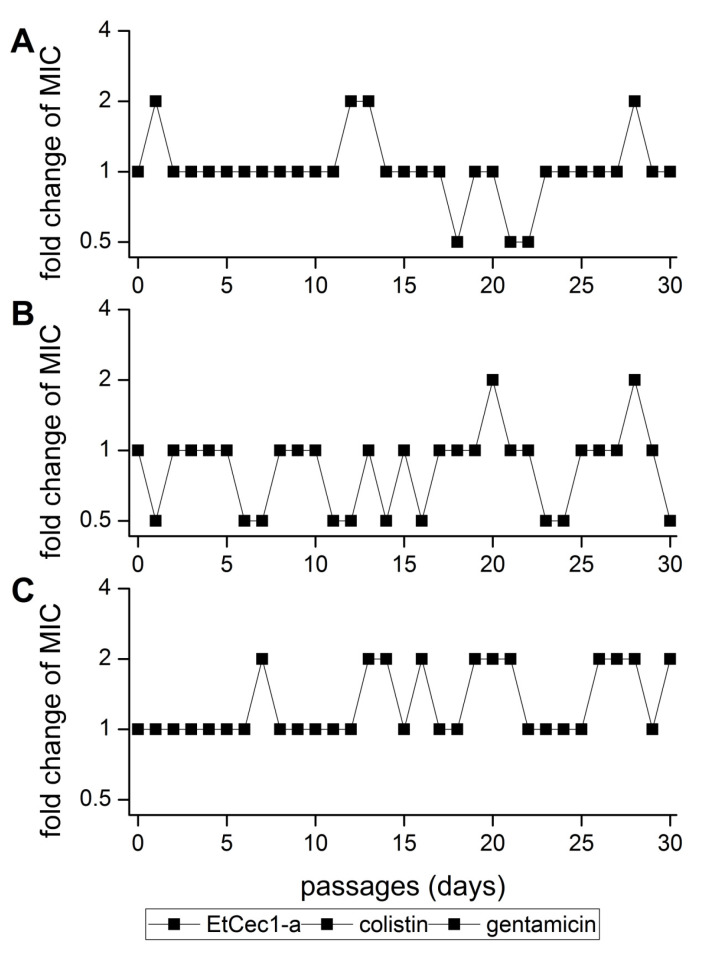
Development of resistance in *E. coli* ATCC 25922 cultures during 30 consecutive days of serial passaging in the presence of sub-MIC concentrations of EtCec1-a. The data show fold-changes in MIC for (**A**) EtCec1-a, (**B**) colistin, and (**C**) gentamicin (*n* = 3).

*E. coli* ATCC 25955 was cultivated in the presence of sub-MIC concentrations of Et-Cec1-a for 30 consecutive days to test for the development of resistance to EtCec1-a. No mutants with a lower MICs as compared to the parent *E. coli* ATCC 25922 strain were generated (Figure 5).

## 4. Discussion

The increasing threat of multidrug-resistant bacteria and the lack of novel antibiotics in the development pipeline have encouraged the screening of AMPs to facilitate the discovery of alternative treatment options. Insects in ecological niches with heavy microbial loads offer a promising source of novel AMPs with potent antimicrobial properties [11,22,23]. We characterized nine AMPs from the rat-tailed maggots of the drone fly *E. tenax*, which thrive in contaminated aqueous habitats. We selected those AMPs displaying the highest differential expression levels upon the injection of bacterial LPS to mount robust immune responses. Initial antimicrobial profiling of the cecropin-like peptides confirmed that their activity was restricted to Gram-negative bacteria, which has been reported for other natural and artificial AMPs representing this family [22,24,25,26]. EtCec1-a was unique in its ability to inhibit the growth of *P. aeruginosa*, with MICs only approximately two-fold higher than the other susceptible strains. This broader activity might reflect its greater hydrophobicity, which is important for antibacterial activity [27,28]. In addition, the strong cationic charge of EtCec1 might facilitate charge-dependent interactions, despite the low negative charge of the *P. aeruginosa* surface [29]. The C-terminally amidated peptides were more potent than their carboxylated derivatives, and this might also reflect their stronger cationic charge [30]. The generally poor performance of EtCec3 confirms that an optimal combination of charge and hydrophobicity is necessary for antimicrobial activity [31]. In contrast to the cecropin-like peptides, the defensin-like AMP EtDef4 was exclusively (but weakly) active against Gram-positive bacteria. Defensins can be active against Gram-positive and Gram-negative bacteria [32,33], but they have more potent effects against Gram-positive species [34,35]. EtDip showed no antimicrobial activity in our initial tests, unlike other diptericin-like AMPs that were reported in the literature [36,37].

The activity of EtCec1-a and EtCec2-a was investigated in more detail against a large panel of multi-drug resistant Gram-negative clinical isolates, revealing no cross-resistance with β-lactams, aminoglycosides, ciprofloxacin, chloramphenicol, or sulfmeracine-trimethoprim. Among today’s clinically approved antibiotics, only the polymyxins (colistin and polymyxin B) display a comparable activity profile [38]. The α-helical cationic AMPs are thought to act through a polymyxin-like mechanism [27,39,40,41,42,43], and our data provide some support for a similar mode of action for the *E. tenax* cecropin-like peptides. These AMPs were completely inactive against the Gram-negative species *P. mirabilis*, *M. morganii*, *S. marcescens,* and *S. fonticola*, which are naturally resistant to polymyxins due to LPS modifications conferring positive charge, such as the attachment of 4-amino-4-deoxy-l-arabinose to the lipid A phosphate groups, and phosphoethanolamine to the core region [16,17,44]. Thus, the binding of cationic peptides is prevented by charge repulsion, possibly explaining the inactivity of other unrelated cationic AMPs against *P. mirabilis*, *M. morganii* and *S. marcescens* [45]. Nevertheless, the mode of action of the *E. tenax* AMPs must differ in some way from that of polymyxins because little or no loss of activity was shown when EtCec1-a was tested against the colistin-resistant isolates of Enterobacteriaceae and *A. baumannii*. Cross-resistance with colistin has been shown for SET-M33L, a tetra-branched artificial peptide, as well as insect cecropins A and B [46], porcine cecropin PI [46], and human cathelicidin LL-37 [47]. AMPs with no cross-resistance to colistin are also known, including structurally nano-engineered antimicrobial peptide polymers (SNAPPs) [48], the artificial peptides WLBU2 and WR12 [47], cecropin A-melittin hybrid peptides [49], and AMPs that were isolated from frog skin [50]. It is unclear why certain AMPs, including the EtCec1-a peptide described herein, show activity against bacteria with acquired colistin resistance. The LPS in the isolates that we tested may be less extensively modified than in naturally polymyxin-resistant species, thus preventing the penetration of polymyxins, but not structurally distinct AMPs [51]. The much higher activity of EtCec1-a when combined with colistin indicates a mode of action distinct from colistin, because combinations of two drugs do not show higher activity when both share the same target. Similarly, we were unable to isolate *E. coli* mutants that were resistant to EtCec1-a, even though serial passaging experiments have shown that both *P. aeruginosa* and *E. coli* develop strong resistance to colistin (>100-fold higher MIC) [51,52], again suggesting a distinct mechanism.

Based on our initial profiling experiments, the *E. tenax* AMPs show little or no antimicrobial activity when presented alone, with the exception of EtCec1-a. The efficacy of innate immunity in insects is enhanced by the co-expression and interaction of numerous AMPs [53,54,55]; hence, the lack of activity observed for individual AMPs is not surprising [23,56]. For example, in the bumble bee *Bombus terrestris*, the AMP abaecin targets the bacterial chaperone DnaK, but in Gram-negative bacteria abaecin only reaches its target when combined with the pore-forming AMP hymenoptaecin, which compromises the cell membrane [57]. All of the *E. tenax* AMPs showed enhanced activity in combination with sub-MIC concentrations of colistin, indicating non-identical modes of action and different intracellular targets. Indeed, even a synergistic relationship was observed in the case of EtDip and colistin. However, the combination of AMP and sub-MIC colistin did not result in lower MIC values, in comparison to colistin. Therefore, these findings need to be validated with colistin-resistant test strains. Only colistin and polymyxin B (but not β-lactams, tetracyclines, aminoglycosides, or rifampicin) increased the activity of the AMPs, which indicated that interactions with membrane-compromising compounds are required in order for these AMPs to reach intracellular targets. Even so, we found that the intrinsically inactive but membrane-compromising nonapeptide polymyxin B did not enhance the activity of any *E. tenax* AMPs [58,59].

The antibacterial activity of AMPs is typically compromised by physiological concentrations of monovalent or divalent ions due to charge repulsion [60,61,62,63]. However, simulated physiological conditions had little or no impact on the activity of the *E. tenax* AMPs.

The systemic application of AMPs is often hampered by their toxicity toward human cells, most likely due to their high net charge and hydrophobicity [64,65]. However, the *E. tenax* AMPs EtCec1-a, EtCec2-a, and EtDip achieved good in vitro therapeutic windows due to the absence of hemolytic, cytotoxic, and cardiotoxic effects. The systemic application of (especially linear) peptides in humans is also hindered by their metabolic instability and short half-life due to extensive proteolysis in the blood, kidney, and/or liver, as well as rapid renal clearance [66,67,68]. We found that EtCec1-a, EtCec2-a and EtDip were metabolically stable (half-life >200 min.), but rapidly degraded in mouse, rat, and human plasma, excluding their systemic application without modification. Various modifications can be carried out to increase the half-life of AMPs [26,45,48,69], but the scope of modifications is limited by the tertiary structure of the AMPs, which strongly influences their activity [70].

In conclusion, given the potency of EtCec1-a as well as EtCec2-a and EtDip in combination with sub-MIC colistin, against multidrug-resistant Gram-negative bacteria, even under simulated physiological conditions, combined with their good in vitro therapeutic windows, these three peptides provide a promising starting point for further development. The derivatives of EtCec1-a could be developed for topical administration or inhalation, whereas EtDip and EtCec2-a may be more useful as scaffolds for the development of adjuvants in combination with polymyxin-derived antibiotics.

## Figures and Tables

**Table 1:** Properties of nine synthetic *Eristalis tenax* antimicrobial peptides (AMPs) *^a^*.

AMP	Sequence ^b^	Size	mol wt (g/mol)	Charge *^e^*	G*^f^*
EtCec1	GFLKKIGKKLEGAVQRTRDATIQTIAVAQAAANVAATAKQG	41	4195.83	+5	−0.07
EtCec1-NH_2_	GFLKKIGKKLEGAVQRTRDATIQTIAVAQAAANVAATAKQ-NH2	40	4137.79	+6	−0.06
EtCec2	GWLRDFGKRIERTGQNIRDATIQTIGIAQEAANVAATLKG	40	4340.86	+2	−0.38
EtCec2-NH_2_	GWLRDFGKRIERTGQNIRDATIQTIGIAQEAANVAATLK-NH2	39	4282.82	+3	−0.38
EtCec3	GFLKKVGKKLEGASDLTRDATIQTIAVAQAAANVAATAKQG	41	4113.67	+3	0.002
EtCec3-NH_2_	GFLKKVGKKLEGASDLTRDATIQTIAVAQAAANVAATAKQ-NH2	40	4055.64	+4	0.01
EtDip	QFNMQGGGSPRQGFDVNANARFPIWQSQNARNSVHGTASYAQHLGGPYGNSRPNFGGGLQFT	62	6654.12	+3.2	−0.83
EtDef1	AACSLGSLLNVGCNSCACAAHCLATRGKNGACNSQRRCVCNK *^c^*	42	4221.86	+5.1	0.12
EtDef4	ATCDLLSFLNVKDAACAAHCLAKGYRGGYCDGRKVCNCRK *^d^*	40	4289.96	+4.1	−0.08
Cecropin A ^g^	KWKLFKKIEKVGQNIRDGIIKAGPAVAVVGQATQIAK	37	4004.77	+6	−0.07
Hymenoptaecin ^g^	HADPQGSLVINGKKPLSGPDRRPSLDVDYHQRVYDRNGMNADAYGGLNIRPGQPAQPHLGVQIQREYKNGFIRGYSQAERGPGGRISPSFGVGGGFRF	98	10676.73	+5.3	−0.86
Defensin 1 ^g^	VTCDLLSAEAKGVKVNHAACAAHCLLKRKRGGYCNKRRICVCRN	44	4830.76	+7.8	−0.17

*^a^* Molecular weights, isoelectric points (p*I*) and net charges were calculated using PepCalc software http://pepcalc.com/. ^b^ Amidation of the C-terminus is indicated by –NH_2_. *^c^* Disulphide connectivity: Cys3-Cys32, Cys13-Cys16, Cys16-Cys18, Cys22-Cys40. *^d^* Disulphide connectivity: Cys3-Cys30, Cys16-Cys36, Cys20-Cys38. *^e^* Net charge at pH 7. *^f^* GRAVY score, total hydropathy values of all the amino acids divided by the size [10]. ^g^ Peptide analogues: cecropin A from *Hyalophora cecropia*; hymenoptaecin from *Bombus pascuorum*; defensin 1 from *Tribolium castaneum* (disulphide connectivity: Cys3-Cys34, Cys20-Cys40, Cys24-Cys42).

**Table 2:** Reference strains and the activity of the *Eristalis tenax* peptides against them. Gray shading = Gram-positive, no shading = Gram-negative; pink shading = acid-fast bacteria and yeast.

	Minimal Inhibitory Concentration (MIC) (µg/mL) *^a,b^*								
	EtCec1 ^c^		EtCec2 ^c^		EtCec3 ^c^		EtDip	EtDef1	EtDef4
Strain	-OH	-NH_2_	-OH	-NH_2_	-OH	-NH_2_			
*Staphylococcus aureus* ATCC 25923	>1024	>1024	>1024	>1024	>1024	>1024	>1024	>128	16
*S. aureus* ATCC 33592	>1024	>1024	>1024	>1024	>1024	>1024	>1024	>128	16
*Staphylococcus epidermidis* ATCC 35984	>1024	nd	>1024	>1024	>1024	nd	>1024	>128	>128
*Enterococcus faecium* DSM 17050	>1024	nd	>1024	>1024	>1024	nd	>1024	>128	>128
*Listeria monocytogenes* DSM 20600	>1024	nd	>1024	>1024	>1024	nd	>1024	>128	16
*Micrococus luteus* DSM 20030	nd	nd	nd	nd	nd	nd	nd	>128	16
*Escherichia coli* D31	16	4	16	4	>1024	128	>1024	nd	nd
*E. coli* ATCC 25922	16	8	64	32	>1024	>1024	>1024	>128	>128
*Klebsiella pneumoniae* DSM 30104	8	4	32	16	>1024	>1024	>1024	>128	>128
*Acinetobacter baumannii* ATCC 19606	16	8	32	8	>1024	>1024	>1024	>128	>128
*Pseudomonas aeruginosa* ATCC 27853	256	32	>1024	>1024	>1024	>1024	>1024	>128	>128
*Proteus mirabilis* DSM 4479	>1024	nd	>1024	>1024	>1024	nd	>1024	>128	>128
*Mycobacterium smegmatis* ATCC 607	>1024	nd	>1024	>1024	>1024	nd	64	>128	32
*Canida albicans* FH2173	>1024	nd	>1024	>1024	>1024	nd	>1024	>128	>128

*^a^* MIC values (*n* = 4) were determined in cation-adjusted Mueller-Hinton broth (CAMB), except for *M. smegmatis* (BHI). *^b^* nd represents values which were not determined. ^c^ Peptides were tested as C-terminally carboxylated (-OH) and amidated (-NH_2_) forms.

**Table 3:** Characterization of the MIC value distribution of four AMPs against the clinical isolates.

Species (no. Isolates)	MIC_50_ *^a^*^,*b*^				MIC_90_ *^a^*^,*c*^			
	EtCec1 -OH *^d^*	EtCec1 -NH_2_ *^e^*	EtCec2 -OH *^d^*	EtCec2 -NH_2_ *^e^*	EtCec1 -OH *^d^*	EtCec1 -NH_2_ *^e^*	EtCec2 -OH *^d^*	EtCec2 -NH_2_ *^e^*
*Escherichia coli* (26)	8	4	32	16	32	8	128	32
*Enterobacter cloacae* (23)	8	4	128	32	128	16	>128	128
*Klebsiella pneumoniae* (21)	32	4	>128	128	128	16	>128	>128
*Salmonella enterica* (10)	32	16	>128	128	32	16	>128	128
*Acinetobacter baumannii* (20)	8	4	16	8	16	8	32	16

*^a^* MIC values were determined in cation-adjusted Mueller-Hinton broth (CAMB). *^b^* Concentration in µg/mL which inhibits the growth of 50% of the tested isolates. *^c^* Concentration in µg/mL which inhibits the growth of 90% of the tested isolates. *^d^* C-terminally carboxylated peptide form. *^e^* C-terminally amidated peptide form.

**Table 4:** Antibacterial activity of the *Eristalis tenax* AMPs under simulated physiological conditions ^a^.

	MIC (µg/mL) *^a,b^*							
	EtCec1			EtCec2			EtCec2-NH_2_	
Strain	CAMB	NaCl	CaCl_2_	CAMB	NaCl	CaCl_2_	CAMB	NaCl
*Escherichia coli* ATCC 25922	16	16	16	64	64	128	32	32
*E. coli* RKI 131/08	16	16	32	32	32	64	16	16
*E. coli* RKI 6A-6	32	nd	nd	256	nd	nd	64	nd
*Klebsiella pneumoniae* DSM 30104	8	32	32	32	16	32	16	32
*K. pneumoniae* RKI 93/10	16	32	32	128	256	256	64	256
*Enterobacter cloacae* RKI 146/09	32	32	64	64	128	128	32	64
*Acinetobacter baumannii* ATCC 19606	16	16	32	32	8	64	8	8
*A. baumannii* RKI 19/09	16	16	nd	16	16	nd	8	16
*Stenotrophomonas maltophilia RKI* 136/09	64	32	nd	512	256	nd	256	512
*K. pneumoniae* RKI 68/16	64	nd	nd	256	nd	nd	128	nd
*K. pneumoniae* RKI 268/15	64	nd	nd	256	nd	nd	64	nd

*^a^* MIC values (n=3)were determined in cation-adjusted Mueller-Hinton broth (CAMB) and in CAMB adjusted to 150 mM NaCl (NaCl) or 1.25 mM CaCl_2_ (CaCl_2_). *^b^* nd represents values which were not determined.

**Table 5:** Activity of *Eristalis tenax* peptides in the presence of sub-MIC concentrations of colistin.

	MIC (µg/mL) *^a,b^*														
	EtCec1 *^c^*				EtCec2 *^c^*				EtCec3 *^c^*				Dip		Colistin
	-OH		-NH_2_		-OH		-NH_2_		-OH		-NH_2_				
Strain	CAMB	Col	CAMB	Col	CAMB	Col	CAMB	Col	CAMB	Col	CAMB	Col	CAMB	Col	CAMB
*Escherichia coli* ATCC 25922	16	4	8	2	64	4	32	4	>1024	4	>1024	4	>1024	4	0.5
*E. coli* RKI 131/08	16	2	4	2	32	2	16	2	>1024	4	>1024	2	>1024	4	0.5
*Klebsiella pneumoniae* DSM 30104	8	2	4	2	32	2	16	2	>1024	4	>1024	4	>1024	4	0.5
*K. pneumoniae* RKI 93/10	16	8	8	nd	128	16	64	8	>1024	>1024	>1024	nd	>1024	>1024	1
*Klebsiella oxytoca*RKI 52/07	16	2	nd	nd	32	2	32	2	>1024	4	nd	nd	>1024	4	2
*Enterobacter cloacae* RKI 146/09	32	4	nd	nd	64	64	32	32	>1024	>1024	nd	nd	>1024	>1024	2
*Acintobacter baumannii* ATCC 19606	16	8	8	4	32	16	8	8	>1024	>1024	>1024	512	>1024	>1024	1
*A. baumannii* RKI 19/09	16	8	4	4	16	4	8	8	>1024	512	>1024	256	>1024	1024	0.5

*^a^* MIC values (n=3) were determined in cation-adjusted Mueller-Hinton broth (CAMB) and CAMB supplemented with 0.075 µg/mL colistin (Col). *^b^* nd represents values which were not determined. *^c^* EtCec1, EtCec2 and EtCec3 were tested as the C-terminally carboxylated (-OH) and amidated (-NH_2_) forms.

**Table 6:** Effects of sub-MIC concentrations of polymyxin derivatives on the activity of *Eristalis tenax* peptides against *Escherichia coli* ATCC 25922.

	MIC [µg/mL] ^a^ for *E. coli* ATCC 25922					
Test Condition ^b^	EtCec1-NH_2_	EtCec2-NH_2_	EtCec3-NH_2_	EtDip	EtDef1	EtDef4
CAMB	16	32	>256	>256	>256	>256
+ Colistin E2 [0.016 µg/mL]	2	2	2	2	4	2
+ PMB [0.016 µg/mL]	2	2	2	2	1	2
+ Colistin E1 [0.016 µg/mL]	4	4	>256	>256	128	8
+ A000500146A [0.0625 µg/mL]	4	4	2	4	128	4
+ A000500059A [0.0625 µg/mL]	16	256	>256	>256	>256	>256
+ A000499933A [0.0625 µg/mL]	16	128	>256	>256	>256	>256
+ A000173039A [0.25 µg/mL]	16	>256	>256	>256	>256	64
+ A000160918 [4 µg/mL]	128	>256	>256	>256	>256	32
+ A000173031A [4 µg/mL]	32	>256	>256	>256	>256	16
+ A000501181A [4 µg/mL]	16	256	>256	>256	>256	>256
+ A000498432A [4 µg/mL]	16	>256	>256	>256	>256	>256
+ A000173033A [4 µg/mL]	16	>256	>256	>256	>256	256
+ A000161246 [4 µg/mL]	128	>256	>256	>256	>256	128
+ PMBN [4 µg/mL]	64	>256	>256	>256	>256	>256
+ A000173380A [4 µg/mL]	64	>256	>256	>256	>256	256

^a^ MIC values were determined in cation-adjusted Mueller-Hinton broth (CAMB) and in CAMB supplemented with sub-MIC concentrations of different polymyxin derivatives. ^b^ values in brackets indicate the concentration; PMB= polymyxin b; PMBM= polymyxin b nonapeptide.

## Supplementary Materials

The following are available online at https://www.mdpi.com/2076-2607/8/5/626/s1. Table S1, Characterization of the clinical isolates; Table S2, Distribution of the MIC values of EtCec1-a in the panel of Gram-negative clinical isolates; Table S3, Distribution of the MIC values of EtCec2-a in the panel of Gram-negative clinical isolates; Figure S1, Heatmap of the 22 *Eristalis tenax* AMPs discovered; Figure S2, Alignment of the Eristalis tenax AMPs with analogues.
