# Supplementary material for: Antimicrobial Peptides from Rat-Tailed Maggots of the Drone Fly Eristalis tenax Show Potent Activity against Multidrug-Resistant Gram-Negative Bacteria
Source: Microorganisms. 2020 Apr 25;8(5):626. doi: 10.3390/microorganisms8050626 (PMC7284870; doi:10.3390/microorganisms8050626)
Supplement: Supplementary file 1 [file microorganisms-08-00626-s001.pdf]

Supplemental

Table S1. Characterization of the clinical isolates from hospitalized patients in Germany (RKI strain collection).

		MIC [µg/mL][a, b]											
**RKI ID**	**Species**	**AMP**	**MSU**	**CTX**	**CAZ**	**GEN**	**KAN**	**AMK**	**OTE**	**CIP**	**SXT**	**MEM**	**CST**
19/09	*A. baumannii*	> 16	> 32	> 16	> 32	> 8	> 32	> 32	> 8	> 64	> 128	> 64	0.5
523/16	*A. baumannii*	> 16	> 32	> 16	> 32	> 8	≤ 2	≤ 2	> 8	> 64	> 128	32	≤ 0.5
584/16	*A. baumannii*	> 16	≤ 2	16	4	1	4	4	8	0.5	8	≤ 0.5	≤ 0.5
596/16	*A. baumannii*	> 16	≤ 2	> 16	> 32	> 8	> 32	> 32	> 8	> 64	> 128	> 64	> 64
271/16	*A. baumannii*	>16	>32	>16	>32	>8	>32	>32	>8	>64	>128	> 64	≤ 0.5
272/16	*A. baumannii*	>16	>32	>16	>32	>8	>32	>32	>8	>64	>128	> 64	≤ 0.5
30/16	*A. baumannii*	>16	>32	>16	>32	>8	>32	>32	>8	>64	>128	> 64	1
70/16	*A. baumannii*	> 16	32	> 16	> 32	> 8	> 32	> 32	> 8	> 64	> 128	> 64	64
32/16	*A. baumannii*	>16	>32	>16	8	>8	>32	>32	4	64	>128	64	2
11/16	*A. baumannii*	> 16	> 32	> 16	> 32	8	> 32	> 32	> 8	> 64	> 128	> 64	1
12/16	*A. baumannii*	> 16	> 32	> 16	> 32	> 8	> 32	> 32	> 8	> 64	> 128	64	≤ 0.5
13/16	*A. baumannii*	> 16	> 32	> 16	> 32	> 8	> 32	> 32	> 8	> 64	> 128	> 64	≤ 0.5
14/16	*A. baumannii*	> 16	≤ 2	> 16	> 32	> 8	4	8	> 8	> 64	> 128	> 64	≤ 0.5
16/16	*A. baumannii*	> 16	> 32	> 16	> 32	> 8	> 32	> 32	> 8	> 64	> 128	> 64	64
230/15	*A. baumannii*	> 16	> 32	> 16	> 32	> 8	> 32	> 32	> 8	> 64	> 128	> 64	≤ 0.5
231/15	*A. baumannii*	> 16	> 32	> 16	> 32	> 8	> 32	> 32	> 8	> 64	> 128	64	≤ 0.5
377/15	*A. baumannii*	> 16	> 32	> 16	> 32	2	> 32	> 32	> 8	> 64	> 128	8	1
3/15	*A. baumannii*	R	nd.	R	R	S	nd.	S	nd.	R	R	32	≤ 0.5
4/15	*A. baumannii*	R	nd.	R	R	S	nd.	S	nd.	R	R	32	≤ 0.5
16/15	*A. baumannii*	R	nd.	R	R	R	nd.	S	nd.	R	R	> 64	≤ 0.5
588/16	*A. pittii*	> 16	≤ 2	16	8	≤ 0.5	≤ 2	≤ 2	2	0.5	8	≤ 0.5	≤ 0.5
146/09	*E. cloacae*	> 16	> 32	> 16	> 32	2	32	≤ 2	4	≤ 0.063	> 128	16	2
33/16	*E. cloacae*	> 16	32	16	4	≤ 0.5	≤ 2	≤ 2	4	0.25	>128	8	≤ 0.5
34/16	*E. cloacae*	> 16	> 32	> 16	> 32	4	> 32	16	8	32	> 128	32	≤ 0.5
113/16	*E. cloacae*	>16	>32	>16	>32	1	4	4	8	4	8	2	≤ 0.5
460/16	*E. cloacae*	>16	>32	>16	>32	>8	>32	32	2	>64	>128	64	≤ 0.5
136/15	*E. cloacae*	> 16	> 32	> 16	> 32	4	> 32	4	8	8	64	> 64	≤ 0.5
141/15	*E. cloacae*	> 16	32	> 16	> 32	≤ 0.5	≤ 2	≤ 2	4	0.125	≤ 4	2	4
210/15	*E. cloacae*	> 16	32	> 16	4	2	8	8	2	≤ 0.063	≤ 4	≤ 0.5	1
243/15	*E. cloacae*	> 16	> 32	> 16	> 32	1	4	≤ 2	2	≤ 0.063	≤ 4	≤ 0.5	4
348/15	*E. cloacae*	> 16	> 32	> 16	> 32	8	> 32	4	8	64	> 128	1	4
506/15	*E. cloacae*	> 16	> 32	> 16	> 32	2	> 32	16	> 8	≤ 0.063	>128	16	1
766/15	*E. cloacae*	> 16	> 32	> 16	> 32	1	4	4	4	≤ 0.063	≤ 4	≤ 0.5	≤ 0.5
76/15	*E. cloacae*	> 16	> 32	> 16	> 32	≤0.5	32	4	> 8	8	> 128	≤ 0.5	≤ 0.5

5/14	*E. cloacae*	> 16	> 32	> 16	> 32	2	> 32	8	> 8	> 64	> 128	4	≤ 0.5
37/14	*E. cloacae*	> 16	4	16	> 32	4	32	≤ 2	8	0.25	> 128	≤ 0.5	≤ 0.5
733/14	*E. cloacae*	> 16	> 32	> 16	> 32	>8	> 32	>32	> 8	> 64	> 128	64	≤ 0.5
287/13	*E. cloacae*	> 16	4	> 16	≤ 2	1	4	≤ 2	2	≤ 0.063	≤ 4	64	> 64
308/13	*E. cloacae*	> 16	4	16	> 32	2	32	≤ 2	4	0.5	> 128	8	≤ 0.5
361/13	*E. cloacae*	> 16	> 32	> 16	> 32	4	32	≤ 2	> 8	0.5	> 128	≤ 0.5	≤ 0.5
466/13	*E. cloacae*	> 16	16	> 16	> 32	4	> 32	≤ 2	4	4	> 128	16	≤ 0.5
546/13	*E. cloacae*	> 16	> 32	16	≤ 2	4	32	≤ 2	> 8	2	> 128	≤ 0.5	2
571/13	*E. cloacae*	> 16	> 32	> 16	> 32	4	16	8	8	≤ 0.063	≤ 4	≤ 0.5	≤ 0.5

**RKI ID**	**Species**	**AMP**	**MSU**	**CTX**	**CAZ**	**GEN**	**KAN**	**AMK**	**OTE**	**CIP**	**SXT**	**MEM**	**CST**
215/15	*E. cloacae*	16	8	≤ 1	≤ 2	1	4	4	2	≤ 0.063	16	≤ 0.5	≤ 0.5
1026/14-1	*E. aerogenes*	> 16	> 32	> 16	> 32	1	4	4	4	0.5	128	16	8
131/08	*E. coli*	> 16	32	≤ 1 (2)	≤ 2	> 8	4	4	> 8	≤ 0.063	> 128	32	0.5
6A-6	*E. coli*	> 16	8	8	16	1	8	8	4	≤ 0.063	≤ 4	≤ 0.5	8
602/16	*E. coli*	≤ 4	nd.	≤ 1	≤ 2	≤ 0.5	nd.	≤ 2	nd.	≤ 0.063	≤ 4	≤ 0.5	16
521/16	*E. coli*	>16	nd.	≤ 1	≤ 2	> 8	32	≤ 2	nd.	> 2	≤ 4	≤ 0.5	8
314/16	*E. coli*	>16	4	≤ 1	≤ 2	2	4	8	4	≤ 0.063	≤ 4	≤ 0.5	8
317/16	*E. coli*	> 16	16	≤ 1	≤ 2	2	8	8	8	1	≤ 4	≤ 0.5	4
330/16	*E. coli*	>16	16	≤ 1	≤ 2	2	> 32	4	>8	>64	>128	≤ 0.5	8
524/16	*E. coli*	>16	nd.	> 16	> 32	≤ 0.5	nd.	≤ 2	nd.	> 2	≤ 4	≤ 0.5	8
525/16	*E. coli*	≤ 4	nd.	≤ 1	≤ 2	≤ 0.5	nd.	≤ 2	nd.	≤ 0.063	≤ 4	≤ 0.5	8
462/16	*E. coli*	>16	>32	≤ 1	≤ 2	1	4	≤ 2	>8	32	8	≤ 0.5	8
443/16	*E. coli*	> 16	>32	>32	>32	4	8	8	>8	>64	>128	4	4
581/16	*E. coli*	>16	32	4	8	1	4	4	nd.	1	32	≤ 0.5	≤ 0.5
582/16	*E. coli*	>16	32	≤ 1	4	1	4	4	nd.	≤ 0.063	32	≤ 0.5	≤ 0.5
583/16	*E. coli*	>16	32	>16	>32	>8	>32	8	nd.	>64	>128	≤ 0.5	≤ 0.5
590/16	*E. coli*	> 16	> 32	> 16	> 32	> 8	8	4	4	0.5	> 128	≤ 0.5	≤ 0.5
592/16	*E. coli*	> 16	> 32	> 16	≤ 2	1	≤ 2	4	> 8	≤ 0.063	8	≤ 0.5	≤ 0.5
593/16	*E. coli*	> 16	> 32	4	4	2	4	4	4	≤ 0.063	16	≤ 0.5	≤ 0.5
607/16	*E. coli*	>16	>32	>16	16	>8	>32	4	>8	2	>128	8	≤ 0.5
513/16	*E. coli*	> 16	32	2	8	2	8	8	1	≤ 0.063	>128	≤ 0.5	≤ 0.5
514/16	*E. coli*	> 16	8	16	>32	2	8	4	4	1	32	≤ 0.5	≤ 0.5
515/16	*E. coli*	> 16	32	8	8	2	8	4	2	64	≤ 4	≤ 0.5	≤ 0.5
95/15	*E. coli*	> 16	> 32	> 16	32	1	> 32	32	2	≤ 0.063	≤ 4	≤ 0.5	≤ 0.5
97/15	*E. coli*	> 16	> 32	> 16	> 32	> 8	> 32	32	> 8	2	≤ 4	≤ 0.5	≤ 0.5
75/15	*E. coli*	R	nd.	R	R	R	nd.	R	nd.	R	R	> 64	≤ 0.5
1023/14	*E. coli*	> 16	≤ 2	> 16	8	1	8	8	2	64	> 128	≤ 0.5	≤ 0.5
867/14	*E. coli*	> 16	> 32	> 16	> 32	≤ 0.5	≤ 2	≤ 2	1	>64	8	8	≤ 0.5
93/10	*K. pneumoniae*	> 16	> 32	> 16	> 32	> 8	> 32	> 32	> 8	> 64	> 128	64	1
600/16	*K. pneumoniae*	8	nd.	> 16	> 32	≤ 0.5	nd.	≤ 2	nd.	> 2	≤ 4	≤ 0.5	32
393/16	*K. pneumoniae*	8	nd.	> 16	> 32	≤ 0.5	nd.	≤ 2	nd.	> 2	≤ 4	≤ 0.5	64
373/16-1	*K. pneumoniae*	16	nd.	> 16	> 32	≤ 0.5	nd.	≤ 2	nd.	> 2	≤ 4	≤ 0.5	32
320/16	*K. pneumoniae*	≤ 4	nd.	≤ 1	≤ 2	≤ 0.5	nd.	≤ 2	nd.	≤ 0.063	≤ 4	≤ 0.5	32

328/16	*K. pneumoniae*	>16	>32	>16	> 32	4	> 32	≤ 2	nd.	> 64	>128	64	64
164/16	*K. pneumoniae*	> 16	> 32	> 16	> 32	> 8	> 32	> 32	> 8	> 64	> 128	> 64	8
138/16	*K. pneumoniae*	>16	4	≤ 1	≤ 2	1	4	4	2	≤ 0.063	≤ 4	≤ 0.5	64
95/16	*K. pneumoniae*	>16	8	≤ 1	≤ 2	≤ 0.5	4	4	2	≤ 0.063	≤ 4	≤ 0.5	> 64
68/16	*K. pneumoniae*	>16	>32	8	8	≤ 0.5	≤ 2	≤ 2	> 8	4	>128	≤ 0.5	64
19/16	*K. pneumoniae*	>16	>32	>16	16	>8	> 32	16	> 8	> 64	>128	≤ 0.5	64
544/13	*K. pneumoniae*	>16	nd.	>16	16	4	nd.	32	nd.	> 64	>128	> 64	16
461/16	*K. pneumoniae*	16	nd.	> 16	> 32	≤ 0.5	nd.	≤ 2	nd.	> 2	≤ 4	≤ 0.5	32
908/15	*K. pneumoniae*	>16	nd.	2	≤ 2	>8	nd.	>32	nd.	> 2	>128	32	32
80/15	*K. pneumoniae*	> 16	> 32	> 16	32	> 8	32	8	> 8	8	>128	≤ 0.5	1
94/15	*K. pneumoniae*	> 16	> 32	> 16	32	> 8	> 32	16	> 8	4	≤ 4	≤ 0.5	≤ 0.5
107/15	*K. pneumoniae*	> 16	> 32	≤ 1	≤ 2	≤ 0.5	≤ 2	≤ 2	> 8	1	≤ 4	1	≤ 0.5
268/15	*K. pneumoniae*	>16	nd.	>16	>32	1	nd.	4	nd.	>2	>128	16	32
577/15	*K. pneumoniae*	> 16	> 32	> 16	4	> 8	32	8	> 8	0.125	> 128	≤ 0.5	≤ 0.5
503/15	*K. pneumoniae*	> 16	> 32	> 16	>32	> 8	> 32	> 32	> 8	4	>128	32	64
229/15	*K. pneumoniae*	> 16	> 32	> 16	>32	>8	>32	>32	>8	>64	>128	16	≤ 0.5
**RKI ID**	**Species**	**AMP**	**MSU**	**CTX**	**CAZ**	**GEN**	**KAN**	**AMK**	**OTE**	**CIP**	**SXT**	**MEM**	**CST**
52/07	*K. oxytoca*	> 16	> 32	> 16	> 32	2	> 32	≤ 2	> 8	64	> 128	32	2
609/16	*K. oxytoca*	>16	>32	>16	16	>8	32	8	1	2	>128	16	1
93/12	*P. aeruginosa*	> 16	> 32	> 16	>32	>8	>32	> 32	>8	0.5	>128	16	≤ 1
628/16	*P. aeruginosa*	> 16	> 32	> 16	4	4	> 32	8	> 8	0.5	> 128	8	1
517/16	*Serratia fonticola*	>16	>32	>16	32	4	4	4	> 8	4	>128	32	> 64
532/14-1	*S. enterica*	> 16	> 32	> 16	> 32	4	> 32	4	> 8	0.5	128	≤ 0.5	8
436/14	*S. enterica*	> 16	> 32	> 16	16	≤ 0.5	≤ 2	≤ 2	2	≤ 0.063	> 128	≤ 0.5	≤ 0.5
270/14	*S. enterica*	> 16	> 32	> 16	8	1	4	≤ 2	4	≤ 0.063	> 128	≤ 0.5	≤ 0.5
205/14	*S. enterica*	> 16	> 32	> 16	16	1	≤ 2	≤ 2	2	≤ 0.063	≤ 4	≤ 0.5	8
583/14	*S. enterica*	> 16	> 32	> 16	> 32	> 8	4	≤ 2	> 8	2	8	≤ 0.5	≤ 0.5
277/14	*S. enterica*	> 16	32	> 16	8	> 8	> 32	4	> 8	0.25	> 128	≤ 0.5	≤ 0.5
419/14	*S. enterica*	> 16	> 32	> 16	8	≤ 0.5	≤ 2	≤ 2	2	≤ 0.063	> 128	≤ 0.5	≤ 0.5
533/14	*S. enterica*	> 16	> 32	> 16	> 32	≤ 0.5	4	4	> 8	0.5	≤ 4	≤ 0.5	≤ 0.5
437/14	*S. enterica*	> 16	> 32	> 16	8	1	4	4	> 8	0.125	> 128	≤ 0.5	≤ 0.5
206/14	*S. enterica*	> 16	> 32	> 16	> 32	> 8	> 32	4	2	≤ 0.063	≤ 4	≤ 0.5	≤ 0.5
729/15	*St. maltophilia*	> 16	32	16	4	>8	> 32	32	>8	8	>128	> 64	> 64
136/09	*St. maltophilia*	8	8	2	≤ 2	2	> 32	4	> 8	4	≤ 4	> 64	8
599/16	*M. morgannii*	>16	>32	>16	>32	2	4	4	>8	16	>128	> 64	> 64
81/15	*M. morgannii*	>16	4	16	16	≤ 0.5	≤ 2	≤ 2	>8	≤ 0.063	≤ 4	≤ 0.5	> 64
93/15	*M. morgannii*	>16	4	4	16	> 8	> 32	≤ 2	>8	16	≤ 4	≤ 0.5	> 64
659/13	*M. morgannii*	>16	nd.	8	8	nd.	nd.	nd.	nd.	≤ 0.063	≤ 4	≤ 0.5	> 64
604/16	*C. freundii*	>16	>32	>16	32	>8	32	4	4	8	>128	> 64	≤ 0.5

[a] AMP, ampicillin; MSU, mezlocillin/sulbactam (0.8 μg/mL); CTX, cefotaxim; CAZ, ceftazidim; GEN, gentamicin; KAN, kanamycin; AMK, amikacin; OTE, oxytetracycline, CIP, ciprofloxacin; SXT,

sulfameracin-trimethoprim; MEM, meropenem; CST; colistin. [b] nd represents values which were not determined.

Table S2. Distribution of the MIC values of EtCec1-a in the panel of Gram-negative clinical isolates.[a]

Species and resistance phenotype[b]	MIC of EtCec1-a (μg/mL)							
(no. isolates)	2	4	8	16	32	64	128	>128
E. coli **(26)**	**1**	**15**	**9**	**1**				
CST^{R} MEM^{R} (1)			1					
CST^{R} (9)		5	4					
MEM^{R} (4)		3	1					
S (12)	1	7	3	1				
E. cloacae **(23)**	**1**	**15**	**3**	**4**				
CST^{R} MEM^{R}(1)				1				
CST^{R} (3)	1	1		1				
MEM^{R} (10)		7	1	2				
S (9)		7	2					
E. aerogenes **(1)**		**1**						
CST^{R} MEM^{R}(1)		1						
K. pneumoniae **(21)**		**6**	**7**	**7**	**1**			
CST^{R} MEM^{R}(6)		1	4		1			
CST^{R} (9)		1	1	7				
MEM^{R} (2)		1	1					
S (4)		3	1					
K. oxytoca **(2)**		**2**						
MEM^{R} (2)		2						
S. enterica **(10)**				**9**	**1**			
CST^{R} (2)				2				
S (8)				7	1			
C. freundii **(1)**			**1**					
MEM^{R} (1)			1					
A. baumannii **(20)**		**10**	**9**	**1**				
CST^{R} MEM^{R}(3)		1	2					
MEM^{R} (16)		9	6	1				
S (1)			1					
A. pittii **(1)**		**1**						
S (1)		1						
P. aeruginosa **(2)**							**2**	
MEM^{R} (2)							2	
S. maltophilia **(2)**						**1**	**1**	
CST^{R} MEM^{R} (2)						1	1	
M. morganii **(4)**								**4**
CST^{R} MEM^{R} (1)								1
CST^{R} (3)								3

***S. fonticola* (1)**								**1**
CST^R MEM^R (1)								1

[a] Table shows the numbers of isolates for which the corresponding MIC value was determined. [b] CST^R, resistant to colistin; MEM^R, resistant to meropenem; S, sensitive to colistin and meropenem.

Table S3. Distribution of the MIC values of EtCec2-a in the panel of Gram-negative clinical isolates.[a.]

Species and resistance phenotype[b]	MIC of EtCec2-a (μg/mL)							
(no. isolates)	2	4	8	16	32	64	128	>128
***E. coli* (26)**		**1**	**8**	**13**	**4**			
CST^R MEM^R (1)					1			
CST^R (9)		1	3	3	2			
MEM^R (4)			1	3				
S (12)			4	7	1			
***E. cloacae* (23)**		**1**	**1**	**5**	**10**		**4**	**2**
CST^R MEM^R(1)							1	
CST^R (3)		1			1		1	
MEM^R (10)				2	5		1	2
S (9)			1	3	4		1	
***E. aerogenes* (1)**				**1**				
CST^R MEM^R(1)				1				
***K. pneumoniae* (21)**				**1**	**7**		**4**	**9**
CST^R MEM^R(6)				1	4			1
CST^R (9)							3	6
MEM^R (2)								2
S (4)					3		1	
***K. oxytoca* (2)**			**1**		**1**			
MEM^R (2)			1		1			
***S. enterica* (10)**					**3**		**6**	**1**
CST^R (2)					2			
S (8)					1		6	1
***C. freundii* (1)**							**1**	
MEM^R (1)							1	
***A. baumannii* (20)**		**1**	**9**	**8**	**1**			**1**
CST^R MEM^R(3)			2	1				
MEM^R (16)		1	7	8				
S (1)								1
***A. pittii* (1)**			**1**					
S (1)			1					
***P. aeruginosa* (2)**								**2**
MEM^R (2)								2
***S. maltophilia* (2)**								**2**
CST^R MEM^R (2)								2
***M. morganii* (4)**								**4**

CST^{R} MEM^{R} (1)	1
CST^{R} (3)	3
***S. fonticola* (1)**	**1**
CST^{R} MEM^{R} (1)	1

[a] Table shows the numbers of isolates for which the corresponding MIC value was determined. [b] CST^{R}, resistant to colistin; MEM^{R}, resistant to meropenem; S, sensitive to colistin and meropenem.

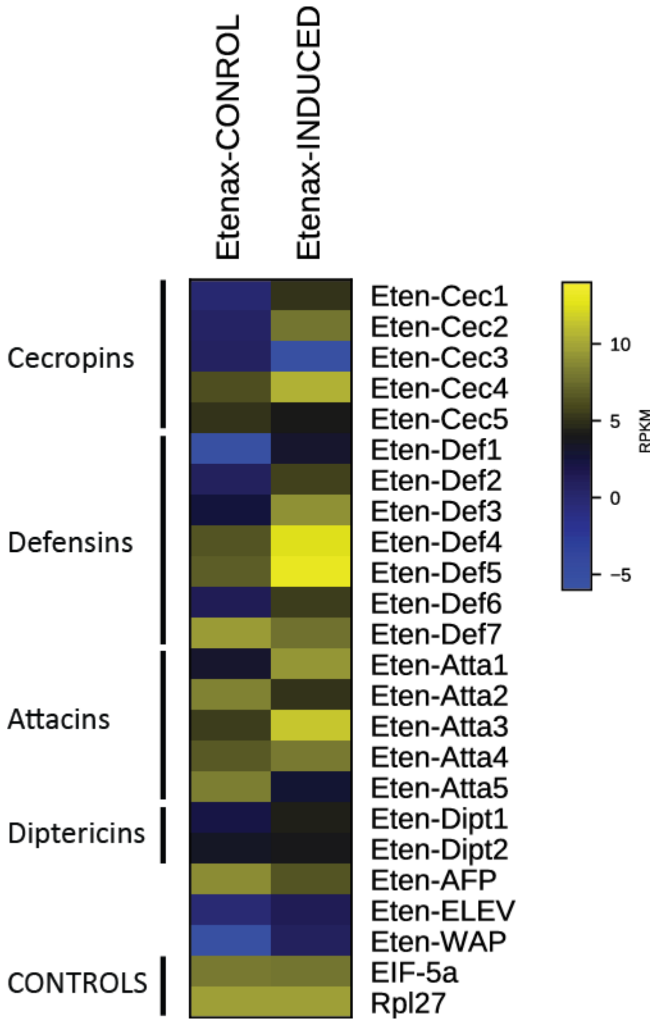

Figure S1. Heatmap of the 22 *Eristalis tenax* AMPs discovered.

```
Cecropin A        1  ---KWKLFKKIEKVGQNIRDGIIKAGPAVAVVGQATQIAK--
EtCec1            1  -GFLKKIGKKLEGAVQRTRDATIQTIAVAQAAANVAATAKQ-
EtCec2            1  -GWLRDFGKRIERTGQNIRDATIQTIGIAQEAANVAATLK--
EtCec3            1  -GFLKKVGKKLEGASDLTRDATIQTIAVAQAAANVAATAKQ-

Defensin 1        1  VTCDLLSAEAKG----VKVNHAACAAHCLLKRKRGGYCNKRRICVCRN-
Et-Def1           1  AACSLGSLLNVG------CNSCACAAHCLATRGKNGACNSQRRCVCNK-
Et-Def4           1  ATCDLLSFLN--------VKDAACAAHCLAKGYRGGYCDGRKVCNCRK-

Hymenoptaecin     1  ---------HADPQGSLVINGKKPLSGPDRRPSLDVDYHQRVYDRNGMNADAYGGLNIRPG
EtDip             1  IN ---------QFNMQG-----GGSPRQGFDVNANARFPIWQSQNARNSVHGTAS-----

Hymenoptaecin    53  QPAQPHLGVQIQREYKNGFIRGYSQAERGPGGRISPSFGVGGGFRF--
EtDip            42  ---IN ----------------------YAQHLGGPYGNSRPNFGGGLQFT
```

Figure S2. Alignment of Eristalis tenax derived peptides to their analogues from other insect species. Shown are similarities of the cecropin-like AMPs to the sequence of Cecropin A from *Hyalophora cecropia,* of the defensin-like AMPs to defensin 1 from *Tribolium castaneum*, and of the diptericin-like AMP to Hymenoptaecin from Bombus pascuorum**.**
